# News and Notes

**Published:** 1911-02

**Authors:** 


### NEWS AND NOTES

### REGULAR MEETING FULTON COUNTY MEDICAL SOCIETY.

REPORTED BY R. R. DALY, M. D.

Dr. Frances Bradley read a paper on "Obligations of the Medical Profession."

Dr. Duncan said this was one of the best papers he had heard in many months. The physician had to deal with all classes of patients and it is a heavy load to carry. Nevertheless, our obligations exist to rich as well as poor, and we must tell them all how to prevent disease and how to train children into healthful development. In the houses of the very poor the problem is grave and with us all. No physician, however long he has practiced, or however short, can cease to care for the indigent. The city provides Ward Physicians, it is true, but often it comes to the family doctor and he must respond.

In Tuberculosis we can limit spread of the disease, even if we cannot cure.

Dr. Hodgson said he criticised only because the essayist was not severe enough upon the doctors. So few doctors take enthusiastic and vital interest in the City's health. The Board of Health ought to have a better backing from us. Our water supply is not well enough guarded. Gas-forming and colon bacilli are in it in our homes.

The smoke nuisance should be abated. The milk supply is incorrect and Dr. Smith should be helped in getting better milk. Noises should be abated. Prisons and Alms Houses should be studied and the conditions remedied.

Dr. Donaldson said that while many of Dr. Hodgson's criticisms were correct, still, as a rule, the physicians of his personal acquaintance were self-sacrificing and alert to the public interests and private needs. They are made better men by these very things. They don't advertise greatly their activities in this direction. Medical men are naturally philanthropic. No other men strive so much to do things that will put them out of a job.

Dr. Simpson congratulated the essayist and urged that the Society take a greater interest in public matters.

Dr. Daly said that the educational problem had not been touched upon in a certain way. There were traditions current in most families that ruled them incorrectly and these must be abolished. The commandment "Honor thy father," etc., was a good ethical principle if the father were worth it, but the ordinary interpretation of that Mosaic injunction does incalculable harm. Children are not consulted when brought into the world and many times are not wanted. The burden of care, protection and love is entirely on the parent and the child owes no duty whatsoever that is not earned by the parent. The child gives love only in return for love and he shouldn't be expected to do anything else. Reasoning from this, the child is not the slave to the father and should not be compelled to follow the benighted and often superstitious hygienic rulings the parent lays down. Just because a father is sick and greatly depressed is no reason for subjecting the children to the infection from the disease that prostrates him, yet, parents insist upon children coming to the tubercular bedside and we have chidren corpses as a result. Hygiene should be handled in the schools so as to make children understand its violations and rebel against them even if the sacred paternal rule received a fatal jolt.

Dr. Kime said he has spoken innumerable times on sociologic lines until he was discouraged. Now he sees rays of hope in the tendency of the profession to tell the public things hitherto concealed from them. He has urged the State Medical Society to take up the matter and has even been criticised for his ideals. The commercialism of the day is a great burden, but even this is lessening. Individually, we are doing more—we know more. If progress continues during the next ten years great good will be done.

Tuberculosis work and venereal diseases are receiving greater attention.

Dr. L. B. Clarke said as a member of the profession that has been maligned, he takes issue with anyone who lays it all to the fault of the medical profession. He compliments Dr. Bradley upon her excellent paper. It is the people who are at fault.

The attempt to eradicate tuberculosis needs money. Should the doctors do it all? The amount of work any doctor has done solely for gratitude is overwhelming. The doctor has been doing the very work advised for fifty years.

Now, what benefit comes to the doctor's family from the thanks of his patients? A good name, it is true, but that is not supporting. The doctor has duties to himself and his family that precludes his giving all his time to philanthropy. The struggle for existence is increasing and that, with the natural strain of interest in patients, wears him out. The people themselves must do more.

Public health per se is not financially supported well enough by the municipality. Grady Hospital is an instance in point. $100,000 for the Hospital is a small part of the $3,000,000 bond issue. He dislikes to see the profession fail to get credit for what it does. Business men laugh at the poor business methods of doctors without understanding why business methods can not be insituted.

Orders are given to put children in bed—there's no fee in that. But these are not obeyed. There's an antagonism between public and doctor. The meetings that Dr. Bradley has instituted do not at present reach the public that needs the education.

Another point, doctors come to these meetings and tell people good things. What business man would use his time in such a fashion, particularly when it militated against his income?

Any medical question arising in this city does not receive good public support.

Dr. Bradley agreed that these meetings do not directly reach the poor people, but the ladies here have spread it and groups of laborers are requesting information. This is ony a means to an end. These ladies are really philanthropic and enthusiastic in spreading the propaganda. They want to get information here from the doctors so that they can do their work better.

Dr. Kime read a paper on "Surgical Cases, (1) Chylous Cyst of the Mesentery, (2) Extensive Abscess of the Abdominal Wall, (3) Cholecystectomy with Complications."

Dr. Battey asked if it were a progressive growth of the multilocular cyst shown.

Dr. Kime said menstruation ceased last March making her apparent time in December, and that the growth had increased since the apparent pregnancy appeared.

Dr. Donaldson said he had a similar case this month and knows how to sympathize with Dr. Kime. His case came for "tapping." There were many symptoms of ascites. Depleting medicines served only to show the presence of the tumor. It was a much easier case than Dr. Kime's because of fewer firm adhesions and the nature of the fluid which came easily through the trochar. The pedicle was small and easily handled. His patient has had no trouble.

Dr. L. P. Stephens said that Dr. Kime's second case illustrates marvelously the resistance a little patient can put up to varied infections. He saw the case and doubted its living at all. He has seen other cases with pneumonia developing in connection with the intestinal intoxication. It teaches us to persist in spite of all complications and difficulties. Child was eight years old.

As to the chylous cyst there is very little literature on the subject. He had a case of a small child from whom he removed about three pints of chyle.

Dr. Kime said the after treatment was proctoclysis medication through rectum and Fowler position. Quinine in large doses is invaluable. Food, peptones, etc., can be given by proctoclysis.

Dr. H. I. Battey related a case in which a lady was confined and delivered of a macerated foetus. Syphilis was suspected from father. Child showed no syphilis. Question arises, can a child get syphilis without infecting mother.

Dr. Ballenger said this is a very deep subject; the tendency of opinion is that the mother has syphilis and probably will give Wasserman reaction positive. It is hard to conceive of the spermatzoon carrying the spirochete to the child and not to the mother. This is under investigation at present by the syphillographers.

Dr. Ballenger reported briefly the histories of eleven patients treated with "606" in whom this remedy had given very satisfactory results. A more complete report will be made later. His experience so bears out that of many others who have seen such wonderful curative effects follow promptly the administration of this remedy.

---

### PROCEEDINGS OF THE AMERICAN PROCTOLOGICAL SOCIETY.--Continued

### "SOME OBSERVATION ON THE PATHOLOGY OF MULTIPLE ADENOMATA."

By Jerome M. Lynch, M. D., of New York City, N. Y.

Who presented the results of his observations on two interesting cases of rectal multiple adenomata. He hoped that others would be sufficiently interested to record and report their own cases, and that our admittedly scanty information on the pathology of this unusual and serious diseased condition would be materially added to.

It was his impression that approximately 46% of recorded cases of this adenomata terminate in cancer and that the ultimate results are commonly fatal; yet the scientific investigation of these tumors has been so comparatively rare and isolated that our actual knowledge of the causes and conditions is lamentably meagre.

Third—That you will meet strong opposition to operative interference by the general practitioner.

Fourth—That you can and should operate at any period of the pregnancy if indicated.

Fifth—That the danger and only danger is in leaving the fissure without operating.

Sixth—That you may and will often have to assume the entire responsibility for the outcome of the operative procedure.

Seventh—That we proctologists should teach on the by-ways and highways of surgery the invariable indication for surgical interference in these unfortunate cases.

---

### A NEW DEVELOPMENT IN LIBRARY WORK.

The Royal Society of Medicine has instituted a new development by which its magnificent library may be useful to its fellows living in any part of the world. Short abstracts are prepared for them gratis, of papers and even of books, on any medical subject, and references to medical literature searched for and checked. This innovation has been warmly welcomed and grateful letters have been received from such remote places as the Chitral Valley, the Sudan and equatorial Africa, where for years men have had their work hindered by the inaccessibility of libraries. Some say that the work is done for them even better than if they had been in London, for they would probably not have been able to devote the many hours required for the research which the machinery of the library procures with a minimum of time and labor.—*Journal American Medical Association,* July 16, 1910.

### "REMARKS UPON CECOSTOMY AND APPENDICOSTOMY."

(With exhibition of a new entero-colonic appendical irrigators.)

By Samuel G. Gant, M. D., New York City, N. Y.

Dr. Gant called attention to the remarkable usefulness of appendicostomy and cecostomy in the direct treatment of bowel diseases and made the point that the latter was preferable in this class of cases and would sooner or later supercede appendicostomy. He also exhibited a new appendical irrigator which could be inserted during operation and which permitted irrigation to be started immediately in aggravated cases of diarrhoea and intestinal auto-intoxication.

Next he showed a new entero-colonic irrigator by means of which the large and small intestines could be irrigated separately or at the same time.

He claimed that this instrument is indicated in the treatment of all forms of enteritis entero-colitis and the different types of ulcerative diseases of the colon and also in the treatment of typhoid fever intussusception, peritonitis and paratic affections of the intestine.

This irrigator he maintained was useful as well for studying the contents of the bowel, intestinal feeding, the direct employment of cathartics, entroclysis and for many other useful and practical purposes.

---

### "A REPORT OF A CASE OF POST-OPERATIVE DELIRIUM."

By Samuel T. Earle, M. D., of Baltimore, Md.

The author stated that while post-operative delirium was quite common before the days of antiseptic surgery, it was due then in the majority of cases to septic infection. The condition is rare now, except when due to shock, and then only as a result of a grave operation.

The minor character of the operation preceding the attack in the present case makes it more interesting, which is doubtless accounted for by the age of the patient.

Case:—Dr. A. T. aged 78, had suffered with hemorrhoids since before the Civil War (1861), but had persistently determined not to be operated upon. Early in May, 1910, they became thrombosed and inflamed, at which time he consented to an operation.

The usual hypodermic of 1-6 of a grain of morphine atropine 1-120, and strychnine sulphate 1-30, was administered prior to the anesthetic. Fearing the effect of ether or chloroform, on account of his age, it was decided to administer a mixture of nitrous oxide gas and oxygen. This mixture did not keep him thoroughly anesthetized, consequently the operation was not completed as quickly as usual and as a result there was more blood lost, which did not exceed two or three ounces.

The operation was completed, he regained consciousness in a few minutes but almost immediately became very excited and delirious. Thinking this might be due to pain, ¼ of a grain of morphine was given at the end of two hours from the time he received the first hypodermic; a third dose was given at 8 P. M., three hours following the second dose. The patient continued very delirious during the night and for three days following. The second and third nights we were able to quiet him for a few hours by hyoscine hydrobromide grain 1-50, and morphine 1-6 administered hypodermically. For the remainder of the first week, the hyoscine hydrobromide 1-50 was sufficient to give him a quiet night, but the delirium continued for one week from the time of the operation, but not nearly to active as during the first few days and with some lucid intervals. His temperature did not exceed 99-½, the first three days but on the fourth day it reached 100.5 and again on the seventh day, for a short time without any apparent cause, otherwise the patient made an excellent recovery, and was able to be about the house in about ten days after operation.

### "APPENDICOSTOMY."

### "A CONSIDERATION OF THE PRESERVATION OF THE BLOOD-SUPPLY OF THE APPENDIX IN THE TECHNIC OF THE OPERATION."

By Frank C. Yeomans, A. B., M. D., New York City, N. Y.

Case—Mrs. X. was operated upon March 21st., 1908, for ulcerative Colitis. While performing the appendicostomy, one of the cecal vessels going to the appendix was punctured and tied. Three days later the appendix sloughed and a fecal fistula formed. The colon healed with irrigations, the fistula closed and the patient is well to-day as regards her bowel. This experience and similar experiences of several colleagues, led the writer to a study of the circulation of the appendix from a surgical standpoint.

Embryology shows the appendix to be the vestige of the original head of the cecum which failed to participate equally in development with the rest of that organ; and at an early period of embryonic life, not possessing a mesentery, derived its sole blood-supply from the cecal vessels. The latter statement is true of the rudimentary and the fetal forms of appendix, even in adults. For all practical purposes the sole blood-supply of the veriform appendix is from the posterior ileo-cecal artery through (a.) its cecal branch, which sends one or more twigs to the appendix, and (b.) its appendicular branch, which runs in the free border of the meso-appendix, sending several (usually (3-5), branches to the appendix. The cecal branch is constant courses along the appendix on its mesenteric side, anastamosing with branches of the appendicular. Dissections of a number of injected subjects, by the writer, demonstrated this arrangement of vessels to be practically invariable. As these vessels are by nature terminal in character, there at once became evident the importance of preserving both branches at operation, if the vitality of the appendix is to be maintained entire.

No trouble is experienced in avoiding the cecal vessels when uniting the cecum adjacent to the base of the appendix to the parietal peritoneum, as they indicate their position by visible pulsation. With the mesentric branches it is different. Most appendices are valciform and one must free the mesentery in order to straighten the lumen sufficiently. There are two ways of accomplishing this:—One is to ligate and cut the mesentery at a point far enough from the base of the appendix that the blood-vessels are preserved to that part of the appendix traversing the abdominal wall. The tip beyond the skin dies and infection is apt to extend between the appendix and abdominal wound, hence this procedure is objectionable.

The other method, here advocated and in practice found successful, preserves the arteries intact and consequently the vitality of the entire appendix. It is accomplished by separating the two layers of the mesentery at its juncture with the posterior mural peritoneum, beginning at its free border, and carefully displacing the cellular tissue with its contained appendicular artery and branches, as far as necessary toward the appendix. The two layers of peritoneum are then divided transversely to the base of the appendix, turned in and sewed, to obliterate the raw space on the posterior abdominal wall. Experience teaches that it is unnecessary to test the potency of the appendix, until the wound has healed, i. e., in 4-5 days.

Further precautions are not to obliterate any arteries by forceps, ligatures, sutures, torsion or tension in fixing the appendix in a position where it does not rest naturally, or by closing the wound too snugly about it.

By following this technic, the operation is without mortality and post-operative leakage of feces and hernia,—the two troublesome sequellae of appendicostomy are avoided· Appendicostomy should continue to grow in favor over cecostomy in all cases where prolonged irrigation of the colon is indicated.

### SOME FACTS THE PUBLIC SHOULD KNOW ABOUT THE EAR AND ITS TREATMENT.

BY LOUIS J. LAUTENBACH, A. M., M. D., PH. D.

PHILADELPHIA, PA.

The physician, to encourage in the public a hopeful attitude toward Ear Disease and Deafness, should emphasize:

1. That one-third of all adults are more or less deaf.

2. That this deafness is frequently avoidable, being usually due to neglect.

3. That this neglect often dates from childhood.

4. That in childhood throat and nose diseases, which are the cause of most ear trouble, are very common.

5. That these throat and nose troubles are in childhood easily cured and ear disease thus avoided.

6. That the adenoid growths and the tonsillar enlargements are common in childhood and easily removed.

7. That the stuffy and occluded nostrils should be properly opened and the child be taught to become a nose breather.

8. That the throat and nose should be thoroughly treated until entirely well.

9. That all ear symptoms should be thoroughly treated as soon as recognized.

10. That if this be done the results will be very favorable and serious ear disease and persistent deafness will be avoided.

11. That the treatment should be persisted in until the ear is cured or benefited to the highest possible degree or until it is absolutely certain that the ear cannot be helped with the means at our command.

12. That ear treatment should be vigorous and perseveringly persistent.

3. That the results of ear treatment are much more favorable than the public have been taught to believe.

14. That no case of deafness is entirely hopeless unless there is destruction of the ear nerve.

15. That each year the increase of knowledge and of new mechanical appliances gives new opportunity to former hopeless cases.

16. That an unfavorable prognosis of a case of deafness (not destruction of the ear nerve), is absolutely certain only as applying to the prognosticator; he cannot of a certainly be sure whether there is not someone of superior knowledge or of greater mechanical skill who can help the case. Nowhere in medicine is this peculiar mechanical skill a greater factor than in the treatment of ear disease.

In short, the public should be taught to avoid ear disease by having their throat and nose troubles cured, not neglected, having ear troubles treated at their onset, and if ear disease has become firmly established to treat it until the best possible results are obtained, using every method that promises relief, never believing that there may not be help, unless there is destruction of the ear nerve. While every case of deafness cannot be cured nearly every case can be helped in a greater or less degree, and no form of ear treatment applied by any skillful specialist can at any time be counted as harmful There are a few cases of ear disease which will in spite of all treatment progress, but fortunately these cases are very few indeed.

1723 Walnut Street.

